# Response time, amplitude, and neural auditory maintenance in individuals with tinnitus: a comparative study

**DOI:** 10.1590/2317-1782/e20240211en

**Published:** 2025-07-28

**Authors:** Christine Grellmann Schumacher, Helinton Goulart Moreira, Denis Altieri de Oliveira Moraes, Larianny Rutzen Lazzari, Michele Vargas Garcia, Dayane Domeneghini Didoné

**Affiliations:** 1 Departamento de Fonoaudiologia, Centro de Ciências da Saúde, Universidade Federal de Santa Maria – UFSM - Santa Maria (RS), Brasil.

**Keywords:** Tinnitus, Event-Related Potentials, P300, Evoked Potentials Auditory, Hearing, Hearing Disorders

## Abstract

**Purpose:**

To verify and compare the response time, amplitude, and neural auditory maintenance of the central auditory pathway in subjects with and without tinnitus disorder.

**Methods:**

This is an analytical, cross-sectional, and quantitative study approved by the Research Ethics Committee. The responses of Long Latency Auditory Evoked Potentials (LLAEP) with verbal stimuli were compared between 16 subjects with tinnitus disorder (Study Group - SG) and 12 subjects without tinnitus (Control Group - CG). The neural response time was evaluated by the latency of the P1, N1, P2, N2, and P300 potentials. The amplitude of these potentials was also analyzed. Neural response maintenance was verified through the duration values of the P300 component. The results were compared between the groups, considering a significance level of 5%.

**Results:**

In the comparison between the groups regarding the latency and amplitude values of the cortical potentials (P1, N1, P2, and N2), no statistically significant differences were observed (p>0.05). However, concerning the latency, amplitude, and duration of the P300 component between the groups, a statistically significant difference was observed for the latency variable, which was greater for individuals with tinnitus disorder (p<0.05).

**Conclusion:**

Individuals with tinnitus disorder have a longer neural response time for the P300 component, suggesting disorganization in central auditory processing.

## INTRODUCTION

The auditory system is made up of sensory structures and central connections whose purpose is to convert sound stimuli into auditory sensations in the cerebral cortex. Any alteration in this system can lead to changes in the various brain areas, which can cause tinnitus^(1,2)^.

Currently, the concept of tinnitus disorder goes beyond the perception of the symptom, but also defines the damage to quality of life due to the perceived impacts^(3)^. Tinnitus is often accompanied by complaints of difficulty understanding speech in noisy environments and cognitive impairment, due to the altered neural signal that is sent through the unsystematic neural connections that tinnitus causes^(2)^. These manifestations can result in problems with attention, concentration, altered sleep, anxiety and depression. This disorder is characterized by a variety of factors that generate and amplify the auditory system, which can be triggered by alterations in the neural organization of the auditory cortex region. These cortical areas are responsible for auditory processing, but are also associated with cognitive aspects such as memory and emotions^(4,5)^.

Based on this definition, Sadeghijam et al.^(2)^ described the theory of chaos, resulting from the dynamic and non-linear functioning of the central auditory system due to tinnitus disorder. This led to the concept of neural deafferentation, an alteration resulting from any reduction in auditory *input* or imbalance between excitation and inhibition, which triggers a compensatory mechanism and becomes an amplification of spontaneous and synchronous neural activity.

In order to assess the functioning of the central auditory pathway, electrophysiological tests can be used to check the functioning and auditory integrity, based on tracings that show the bioelectric activity of the auditory pathways after acoustic stimulation^(6-8)^. Among the existing electrophysiological tests for assessing tinnitus are Long Latency Auditory Evoked Potentials (LLAEP)^(9,10)^.

The LLAEP reflects the functionality of central auditory processing through the latency and amplitude of the P1, N1, P2, N2 and P300 potentials. The P1, N1 and P2 components are independent of the subject's response and are related to the integrity of the auditory pathway, neural coding, perception, stimulus detection and auditory discrimination^(7)^. The N2 is considered a mixed component, as it is triggered by both endogenous and exogenous factors, contributing to auditory stimulus discrimination activities and reflecting the individual's attentional factors^(8)^. The P300 is considered an endogenous potential, as it depends on the individual's response and, from this, provides information on auditory discrimination, attention and recent memory^(7)^. Furthermore, this potential is considered a biomarker for tinnitus disorder, since this symptom can cause changes in auditory and cognitive neural functioning^(9-12)^.

Some researchers^(10)^ have reported changes in the latency and amplitude values of LLAEP in subjects with tinnitus, due to the phantom focus of attention that exists in the symptom, recruiting cognitive capacity as a competitive stimulus that attracts attention to the symptom^(7,9,11)^. In this way, these individuals may show changes in processing time and neural recruitment^(13)^. Furthermore, alterations in the P300 component may be observed in this population due to the changes in non-auditory regions mentioned above, which participate in the generation of this component^(12,14)^. However, researchers have identified that mild chronic tinnitus in individuals with normal hearing does not interfere with divided auditory attention and verbal auditory memory^(11)^, highlighting the differentiation between the concept of tinnitus and tinnitus disorder and its influence on cognitive aspects^(3)^.

In addition to latency and amplitude measurements, another observable parameter in the electrophysiological tracing is the duration of the P300 component. Hall^(15)^ defines duration as the difference in milliseconds between the rise and fall of this wave, i.e. how many milliseconds the P300 wave lasts, measured from the beginning of the peak to its end. The duration of the P300 can be important data for verifying the functionality and association of auditory and cognitive areas, since this measurement can be associated with the number of neurons and synapses involved in generating and maintaining auditory activity during the generation of this potential. Despite this, there is no description in the literature of the analysis and measurement of this variable in subjects with tinnitus disorder.

Based on the possibility of central auditory alterations observed in the LLAEP components in patients with tinnitus disorder and the scarcity of studies analyzing the duration of the P300 component, this study aimed to verify and compare the response time, amplitude and neural auditory maintenance of the central auditory pathway in subjects with and without tinnitus disorder.

## METHODS

This is an analytical, cross-sectional and quantitative study, approved by the Research Ethics Committee under number 64696022.1.0000.5346. The sample was collected by convenience. Contact was made through the social networks of the audiology service where the research was carried out. The research procedures were carried out in a school clinic at the Federal University of Santa Maria, from June 2023 to January 2024. The study followed the norms and guidelines of Resolution 466/12 of the National Health Council and all individuals who consented to take part in the research signed the Informed Consent Form (ICF), which described the procedures, risks, benefits and data confidentiality.

The eligibility criteria established for subjects without and with tinnitus disorder were:

- aged between 18 and 55;
- both sexes;
- Brazilian Portuguese as a mother tongue;
- right hand preference;
- educated - more than twelve years of schooling;
- hearing thresholds of up to 19 dBHL at frequencies from 250Hz to 8000Hz;
- type “A” tympanometric curves, according to Jerger et al.^(16)^;
- contralateral acoustic reflexes present at normal levels bilaterally according to Jerger et al.^(16)^;
  -abnormality in the Brief Neuropsychological Assessment Instrument-NEUPSILIN;
- integrity in the Brainstem Auditory Evoked Potential (BAEP), according to Webster (2016);
- normality in the Central Auditory Processing (CAP) assessment.

The exclusion criteria were:

- musicians or those exposed to musical practice;
- have a diagnosed and/or obvious neurological and/or psychiatric complaint or impairment;
- complaints of dizziness or continuous exposure to noise.

For subjects with tinnitus disorder, the following inclusion criteria were added:

- Subjective tinnitus bilaterally, with no evidence of a vascular component (of the pulsatile type);
- complaints about the impact on quality of life;
- a score on the Visual Analog Scale (VAS) of at least 4 points, considered moderate discomfort;
- symptom perception time greater than six months.

The following exclusion criteria were adopted for subjects with tinnitus disorder:

- perform another intervention for the symptom;
- use continuous medication or pharmacological treatment for tinnitus;

The study was divided into two stages: on the first day, the assessment procedures were carried out, including a basic audiological assessment, central auditory processing tests and an assessment to measure tinnitus (for the initial composition of the sample), with a total duration of one hour and 30 minutes. On the second day, the research procedures were carried out, including the LLAEP (for data analysis), taking a total of one hour and 30 minutes to complete. These procedures were always carried out in the same order in both groups.

The procedures were carried out to ensure normal peripheral hearing, the integrity of structures up to brainstem level and normal central auditory processing skills, ensuring that there was no influence on the LLAEP findings. To guarantee the quality of the procedures carried out, all the equipment used in this study was calibrated.

To compose the sample, the following procedures were carried out: semi-structured anamnesis, basic audiological assessment, visual inspection of the external acoustic meatus, pure tone audiometry (PTA), logoaudiometry and acoustic immittance measurements, in order to select the individuals who met the eligibility criteria assigned in this study.

A total of 85 subjects were seen, 50 (58.82%) of whom were men and women complaining of tinnitus. Of these, six (12%) were excluded due to otitis and/or Eustachian tube dysfunction, 10 (20%) due to neurological and/or psychiatric diseases, six (12%) because they were already undergoing treatment for tinnitus and 12 (24%) because they had hearing loss diagnosed between the frequencies of 250 and 8000Hz. As a result, the study group consisted of 16 individuals. The control group included 35 subjects who did not complain of tinnitus. Of these, 13 (37.14%) had altered central auditory processing and 10 (28.57%) had hearing loss, making up 12 subjects in the group without tinnitus disorder.

The CAP tests were selected with the aim of covering the minimum battery suggested according to the recommendations of the *American Speech-Language-Hearing Association - ASHA* ^(17)^, in which an altered test was considered to be a Central Auditory Processing Disorder (CAPD), based on the study that analyzed the central auditory processing of subjects with and without tinnitus^(18)^. All the tests were carried out at 40 dBSL above the tritone mean, as it was possible to use the same technique as the SRPI, since the subjects had no reduction in peripheral hearing acuity^(19,20)^.

For the CAP assessment, the tests were carried out in an acoustically treated booth, using supra-aural headphones, model TDH39, brand Telephonics, a two-channel audiometer, model AD629B, brand Interacoustics, connected to a *notebook* to direct the assessments. The following assessments were carried out:

Dichotic Digits Test (DDT)^(20)^: A test that assesses figure-ground ability for verbal sounds, with the binaural integration stage being investigated. The stimuli were presented binaurally and normality values equal to or greater than 95% were considered^(20)^.

Auditec Frequency Pattern Test (TPF)^(20)^: A test that assesses the ability to order non-verbal sounds in time. The stimuli were presented monaurally and a reference value of 86.6% or more was used^(20)^.

Masking Level Difference (MLD)^(20)^: A test that analyzes the auditory ability of binaural interaction and selective attention. The stimuli were presented binaurally and a score of 8 dB or more was considered normal^(20)^.

Gaps in Noise (GIN)^(21)^: A test that assesses temporal resolution skills. The stimuli were presented monaurally. Band 1 was used in both ears and a normality criterion of 6ms was adopted^(21)^.

Speech in Noise (SR)^(22)^: A test that assesses the ability to close one's hearing to verbal sounds. The stimuli were presented ipsilaterally with a signal-to-noise ratio of + 5dB. The standard adopted was 70% correct in both ears^(22)^.

To ensure that neuropsychological abilities were preserved, NEUPSILIN^(23)^ was used. This protocol analyzes eight main cognitive functions: temporal-spatial orientation, concentrated auditory attention, visual perception, memory, arithmetic skills, language, praxis and executive functions. For the study, the total score of the tasks was considered to be within the normal range suggested by the authors^(23)^.

*Tinnitus* disorder was measured and assessed by means of a *tinnitus* anamnesis, with questions about general health history and factors influencing the symptom, and by means of the Visual Analog Scale (VAS) and the *Tinnitus Handicap Inventory* (THI).

The VAS was printed out and numbered from 0 to 10, with one end of the line meaning “no tinnitus” and the other “the worst tinnitus imaginable”. The individual was asked to rate the annoyance of tinnitus at the time of data collection. A scale score of 4 or more points was considered moderate annoyance^(24)^.

The THI questionnaire was applied to all individuals with tinnitus disorder and its aim was to check quality of life and classify it into degrees according to the test score^(25)^. The questionnaire has 25 questions and each answer has a value to be counted. At the end of the test, the score was added up and classified according to the degree of discomfort and impact on quality of life.

For auditory evoked potentials, we used the *Smart EP* two-channel equipment *from Intelligent Hearing Systems (IHS).* Initially, the skin was cleaned with *Nuprep* exfoliating gel, after which the electrodes were attached, with the ground electrode in Fpz; the active electrode in Fz for brainstem auditory evoked potentials (BAEP) and in Cz (cranial vertex) for LLAEP. The reference electrodes were attached to the right and left earlobes (A2 and A1). Neurobase electrolytic paste was used and the electrodes were fixed to the subjects' skin with micropore tape at specific points for each potential. The inter-electrode electrical impedance was kept equal to or less than 3kΩ during all the evaluations. To avoid further electrical interference and/or muscle artifacts, the light in the room was turned off and the subjects were instructed to close their eyes, remain relaxed and avoid movement.

BAEP was performed monaurally, with a rarefied polarity click stimulus, at an intensity of 80 dBnHL, a speed of 27.7/s, a gain of 100K, a filter of 100-3000Hz, a recording window of 12ms, an electroencephalogram (EEG) window of 31% and 2048 stimuli, and two collections were made to check the reproducibility of the waves. The criterion for identifying the integrity of the auditory pathway was the presence of waves I, III and V and absolute latencies and interpeak intervals within normal standards. The values were analyzed using the study by Webster as a reference^(26)^, taking into account two standard deviations (2 SD).

As a research procedure, the Long Latency Auditory Evoked Potential with verbal stimuli (LLAEP-verbal) was performed^(27)^. This was done binaurally, using insert earphones at an intensity of 80 dBnHL, with 300 verbal stimuli, produced from the syllables /ba/ and /di/, representing the frequent stimulus (80% of the time) and the rare stimulus (20% of the time) respectively, based on the oddball paradigm. At first, the test was simulated by speaking the /ba/ and /di/ sequences so that the subjects would understand how the assessment worked. Next, the subjects were instructed to mentally count the stimulus /di/. At the end of the test, the examiner asked how many stimuli had been counted in order to compare them to the total number of targets presented by the equipment, ensuring that the individual had carried out the proposed activity correctly.

**Figure 1:**
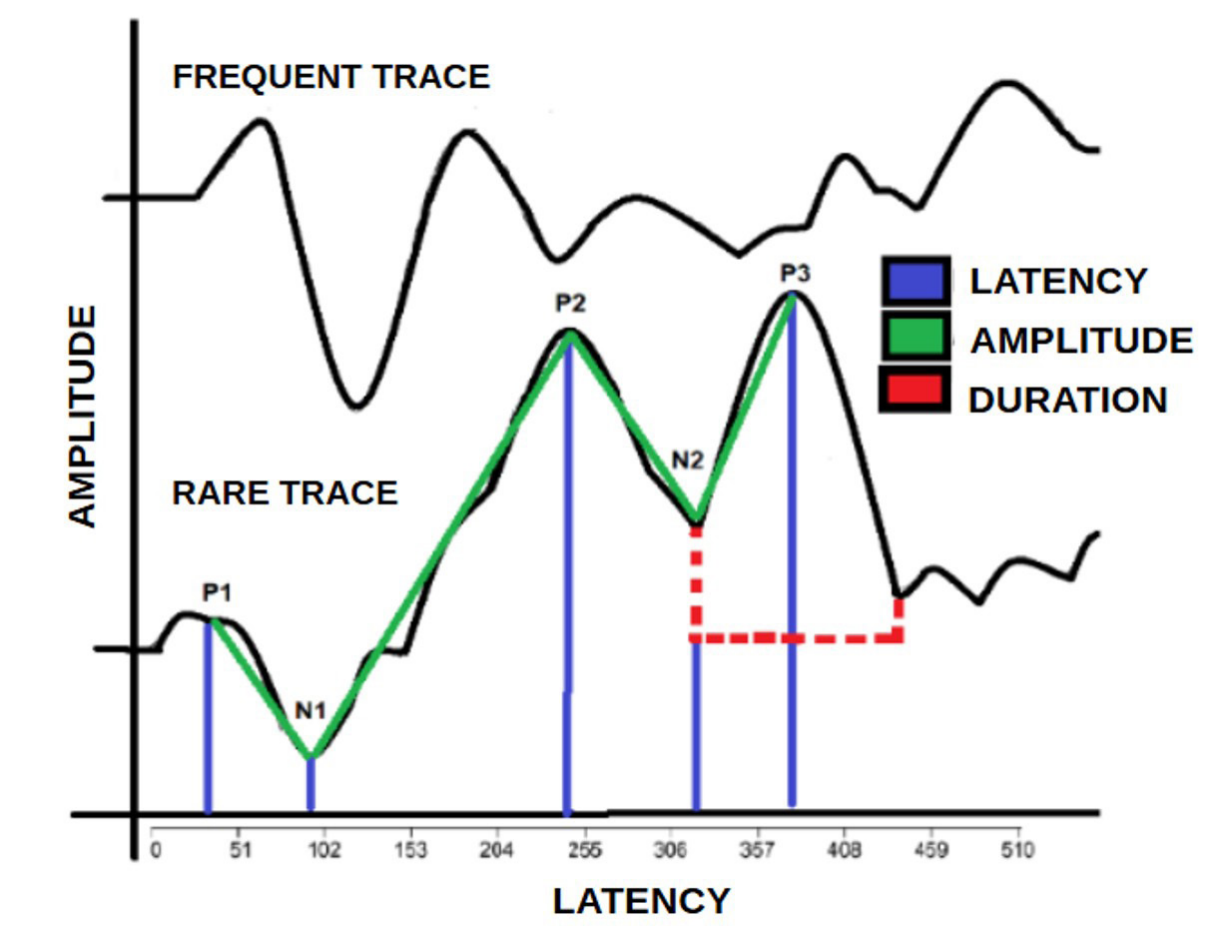
Marking of LLAEP-verbal waves on the r-trace

**Source**: Image created by the authors, based on the marking of the components in the rare tracing

The evaluation was carried out at a speed of 1.10/s, recording window of 510ms, gain of 100K, filter of 100-3000Hz and electroencephalogram (EEG) window of 31%. The latency (ms) and amplitude (µs) of the P1, N1, P2, N2 and P300 waves were marked on the rare tracing^(28)^. We also marked the duration of the P300 wave, measured in milliseconds, from the rise to the fall of the potential (Figure 1), subtracting the final latency from the initial one. The N1 and N2 components were only marked when the amplitude was negative.

Figure 1 shows a representative image of the LLAEP-verbal, showing the measurement of the parameters analyzed (latency, amplitude and duration).

After meeting the inclusion criteria, the participants were divided into two groups: the Study Group (SG), made up of subjects with tinnitus disorder, and the Control Group (CG), made up of individuals without tinnitus disorder.

Table 1 shows the description of the participants in each group in terms of gender and age. The average VAS score was 6.81 points (minimum= 4/maximum= 10) in the tinnitus disorder group. In addition, the THI showed an average score of 51 points (moderate degree) (minimum= 20/maximum= 94).

**Table 1:** Descriptive data of the sample

**Variable**	**CG**	**SG**	**p-value**
n	12	16	
Gender M/F	3 / 9	6 / 10	0.496
Age (average)	23.83±4.69	35.75±13.48	0.004
Education	13.25±2.70	12.31±1.54	0.944

Mann-Whitney U test used

**Caption:** N = sample size; M = male; F = female; CG = control group; SG = study group

The data was entered into an *Excel* spreadsheet and the statistical analysis was carried out in the R *software* ^(29)^ by a professional in the field. Initially, the Shapiro-Wilk test was used to verify the normality of the data and, consequently, the choice of statistical test. Then, the ears in each group were compared using the T-test for paired samples. The analysis between the groups was carried out using the T-test for independent samples. A 5% significance level was used for all analyses.

## RESULTS

Initially, the results were analyzed between the right and left sides intra-groups. There was no statistically significant difference (p>0.05) between the sides for all the variables analyzed. Therefore, the mean values were used to compare the groups.

There was no statistically significant difference when comparing the latency and amplitude values of the P1, N1, P2 and N2 potentials between the groups (Table 2).

**Table 2:** Comparison of latency and amplitude values of potentials P1, N1, P2 and N2 between groups

**Variable**	**Group**	**n**	**Mean ± SD**	**Min - Max**	**Difference**	**P-value**
Latency P1	CG	12	69.5 ± 12.21	54 - 95	4.59	0.362
	SG	16	64.91 ± 13.51	45 - 85		
Amplitude P1	CG	12	4.3 ± 1.59	1,0 - 6,9	0.07	0.938
	SG	16	4.23 ± 2.67	0.3 – 10.9		
Latency N1	CG	12	114.21 ± 10.49	94.0 – 148.0	0.43	0.929
	SG	16	113.78 ± 13.68	89.0 – 145.0		
Amplitude N1	CG	12	-5.02 ± 1.47	1.9 – 8.5	0.5	0.686
	SG	16	-5.52 ± 4.58	4.9 – 13.4		
Latency P2	CG	12	179.88 ± 14.61	156.0 – 205.0	11.34	0.145
	SG	16	191.22 ± 2283	141.0 – 239.0		
Amplitude P2	CG	12	4.4 ± 3.13	0.9 – 11.9	0.15	0.893
	SG	16	4.24 ± 2.85	0.4 – 10.9		
Latency N2	CG	12	249.82 ± 33.72	205.0 – 304.0	14.15	0.318
	SG	16	263.97 ± 36.56	145.0 – 322.0		
Amplitude N2	CG	12	-2.98 ± 2.14	0.4 – 7.7		0.257
	SG	16	-1.87 ± 2.61	3.3 – 7.5	1.1	

T-test for independent samples used

**Caption:** n = sample size; L = latency; A = amplitude; CG = control group; SG = study group

When comparing the latency, amplitude and duration values of the P300 component between the groups (Table 3), there was a statistically significant difference for the latency variable, which was higher for individuals with tinnitus disorder. The amplitude and duration variables showed a significance value of less than 10%, indicating a possible tendency towards differences between the groups.

**Table 3:** Comparison of the latency, amplitude and duration values of the P300 potential between the groups

**Variable**	**Group**	**n**	**Mean ± SD**	**Min - Max**	**Difference**	**P-value**
**Latency P3**	**CG**	12	306.91 ± 40.99	226.0 – 373.0	34.53	**0.021**
	**SG**	16	341.44 ± 31.91	256.0 – 418.0		
Amplitude P3	CG	12	5.17 ± 3.53	1.3 – 9.6	1.93	0.085
	SG	16	3.24 ± 2.07	0.7 – 9.1		
Duration P3	CG	12	132.17 ± 42.09	63.0 – 215.0	29.07	0.076
	SG	16	103.09 ± 40.63	21.0 – 186.0		

T-test for independent samples used

**Caption:** n = sample size; L = latency; A = amplitude; CG = control group; SG = study group

**Figure 2:**
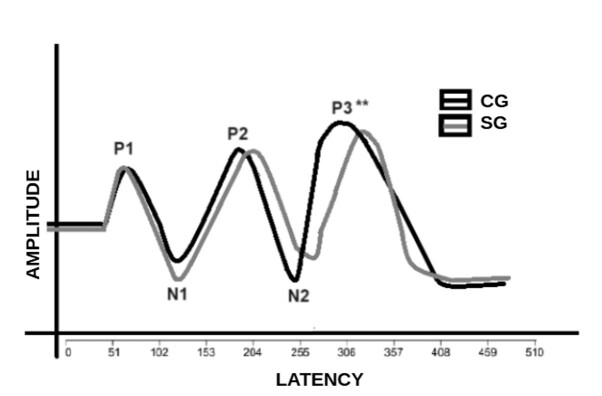
Graphical representation of the LLAEP-verbal between subjects with and without tinnitus disorder

**Source:** Created by the researchers

Figure 2 shows a graphical representation of the verbal LLAEP for the control and tinnitus groups, with a statistically significant difference only for the P300 latency.

**Caption:** CG: control group; SG: study group; ** Statistically significant difference for P300 latency

## DISCUSSION

Auditory evoked potentials are used to detect neuronal activity and the activation of auditory fibers, and understanding the neurophysiology of tinnitus is extremely important for the therapeutic process. The use of the LLAEP as a biomarker in tinnitus patients has brought major findings regarding the influence of the symptom on central auditory aspects^(10)^. This potential has increasingly demonstrated its clinical influence, revealing cognitive abilities and auditory skills through the components assessed and their generating sites. As such, it has become an ally in clinical assessment to measure the impact of tinnitus, taking into account the manifestation of the symptom and changes in the auditory-cognitive-emotional-executive functioning of multiple brain areas.

This study is in line with recent international research, such as that by Cardon et al.^(30)^, which aimed to develop a model for detecting cases of tinnitus based on auditory evoked potentials as biomarkers for diagnosis and monitoring. In the same way as Morse and Vander Werff^(31)^, this study sought to observe the responses of Cortical Auditory Evoked Potentials (CAEP), evaluating cortical auditory onset *versus* displacement responses. These citations demonstrate the unquestionable relevance of the subject, exposing the findings of LLAEP in subjects with tinnitus disorder.

A major difference in this study is that it measures more than latency, aiming to demonstrate the neural responsiveness (response amplitude) of the components and the duration of the P300 component. Studies have shown the importance of analyzing duration in auditory evoked potentials. A pioneering study carried out in the elderly demonstrated the importance of this measure, in which the P300 potential and *Mismatch Negativity* were analyzed^(32)^. This analysis was used to measure the time to auditory discrimination, since the duration involves the initial and final latency of the P300. Therefore, taking into account the impacts of tinnitus perception, observing its neural maintenance for counting - memory - of the stimulus is extremely important.

It is worth noting that recent studies aimed at measuring neuroplastic changes in the central auditory pathway, especially in auditory cortical regions, usually had non-homogeneous populations or other associated variables, whether related to ageing, pathologies or sound perception disorders^(33,34)^.

Thus, it is believed that, in this study, the difference in P300 latency between the groups was due to the probable neural disorganization of patients with tinnitus disorder, since the variables of education and gender did not show statistically significant differences when comparing the groups, i.e. it is assumed that the cognitive level of the patients is similar. It is believed that, despite the difference in age between the groups, this variable did not influence the P300 latency results. One study highlighted significant changes in latency values from the age of 60^(35)^, an age group not included in this study. In addition, researchers^(36)^, aiming to characterize the auditory pathway of the elderly, have shown that the P300 is less sensitive to changes due to age, but rather due to specific characteristics^(37)^. Thus, it could be suggested that the potential is influenced by the perception of the symptom and the neural maladjustment caused by tinnitus, and not necessarily by age, given that only highly educated adults were included.

With regard to cortical auditory potentials, the data from this study showed no differences between the groups for the P1, N1, P2 and N2 potentials, as has already been observed in other studies^(38)^. Some researchers have only observed differences in the amplitude of the P2 component, which may be related to the influence of tinnitus on the discrimination of the acoustic stimuli presented.

From the analysis of the results of the P1, N1, P2 and N2 potentials, described in Table 1, it was observed that individuals without and with tinnitus have similar functionality of the cortical auditory regions. A recently published systematic review^(9)^ showed that tinnitus patients had alterations in the functioning of the central auditory pathways, with changes in the latency and/or amplitude values of event-related long latency potentials. However, the authors point out that these changes are commonly associated with the severity of the tinnitus, the site of the lesion and the capacity for changes after interventions. Such statements about these changes are still incongruous in the specialized literature, since studies have shown relevant changes and other similarities in the auditory cortical functioning of patients without and with tinnitus^(9,10,31)^.

Individuals with tinnitus often have alterations in CAP skills. However, in the present study, the participants with the perception of the symptom had no complaints and their skills remained within the reference standards. Thus, this may be another aspect that justifies the similar findings of the P1, N1, P2 and N2 components between the control group and the study group, as these potentials represent the central functionality of various auditory skills, which, when altered, can influence the latency and amplitude variables of these components^(10,18)^.

The findings of this study showed an alteration in the latency of the P300 potential when comparing subjects without and with tinnitus disorder. Subjects with tinnitus disorder have a deafferentation of stimuli in the thalamic region, with hyperactivation in the parietal region and temporal gyrus, due to the pathophysiological mechanism^(39,40)^. Thus, alterations can be expected in the central regions, which are the sites that generate the P300 component. This finding has also been observed in other studies, in which changes in P300 latency values were observed^(14)^. A recent systematic review with meta-analysis^(30)^ highlighted that the P300 is the main biomarker for subjects with tinnitus among the LLAEP, highlighting the importance of the finding in relation to the P300 latency in the present study.

It is worth noting that research^(41)^ with subjects who have had tinnitus for less than 10 months has shown no significant differences in the characteristics of the P300, highlighting the relationship between the differentiation of tinnitus and tinnitus disorder proposed by De Ridder^(3)^, i.e. the length of time the symptom has been perceived is related to greater neural disorganization and other cognitive and psychological effects. In the present study, only subjects with tinnitus disorder, characterized by moderate annoyance, were included, suggesting that there is a modification of the central auditory pathway in subjects with this characteristic.

In general, tinnitus disorder interferes mainly with aspects related to attention, memory, speech perception and directly with quality of life, i.e. the individual's behavior. The P1, N1 and P2 potentials are cortical, endogenous and “automatic”. P2 is considered important for observing thalamic dysrhythmia or central inhibition^(33)^. However, the sensitivity of this component for subjects with tinnitus is controversial in the literature. It is therefore believed that the N2 and P300 components can better reflect the individual's behavior related to the symptom^(38)^. Therefore, differences in these potentials in subjects with tinnitus are more common, as was observed in the P300 latency in this study (Figure 2).

The amplitude of the P300 is intrinsically related to neuronal activation to respond to the activity proposed to generate this component. Subjects with tinnitus, due to thalamic arrhythmia, may have inhibited attention to the external stimulus, generating a lower amplitude in this component^(9)^. Although no statistically significant difference was found in this study, there was a possible tendency towards differences between the groups (p-value below 10%), with lower values in patients with tinnitus disorder. The great variability in amplitude values and the small sample size may have contributed to these results not being statistically significant.

The duration of the P300 component is associated with the maintenance of neuronal activity during the auditory task^(9,37)^. In this study, as with amplitude, there was a possible statistically significant trend between the groups, and the results were lower in the tinnitus group, but were not significant. These data are noteworthy because a reduced duration was observed in subjects with tinnitus disorder and normal auditory processing, suggesting that the perception of this symptom may have a direct influence on the neuronal functioning of these subjects. It is believed that a larger sample size could show significant differences between the individuals assessed. There have been no studies describing the duration of the P300 potential in subjects without and with tinnitus disorder, which is unprecedented in this study.

The literature suggests using different types of stimuli to assess LLAEP in individuals complaining of tinnitus, including the speech stimuli used in this study. These stimuli are ideal for verifying the functionality of the neural bases of speech detection and discrimination, contributing additional information on the processing of complex signals^(10,27)^. It is important to note that in this type of assessment, tinnitus can become a third auditory stimulus^(11,42)^, influencing LLAEP components, especially the P300, which is associated with aspects of auditory discrimination, attention and memory. Thus, the increase in latency values and reduction in amplitude and duration values of the P300 of the subjects in this study can be explained by this possible attentional shift, i.e. neural recruitment and maintenance for the rare stimulus in the LLAEP assessment became more difficult due to the presence of tinnitus, directly impacting on the speed of neural processing.

The fact that this study showed a change in auditory response time and suggested alterations in neural recruitment and maintenance as evidenced by the latency, amplitude and duration of the P300 component brings a new perspective to the clinical use of LLAEP in subjects with tinnitus disorder, highlighting the importance of the aforementioned analyses to verify the maintenance of neuronal activity during the auditory and cognitive process in the electrophysiological evaluation.

Therefore, it may be useful to apply this knowledge to tinnitus therapies based on *neurofeedback*, in order to maximize the ability to divert attention from tinnitus, as well as the use of cognitive-behavioral therapies to divert the focus of attention from the symptom. Another relevant aspect is related to the analysis of the P300 potential as a possible biomarker in the process of rehabilitating the symptom, evaluating the aspects before and after the intervention. Considering the cortical and cognitive areas represented by the LLAEP, stimulation of the auditory-cognitive pathway with the aim of promoting central reorganization could be an effective therapeutic strategy, since this is a neuroplasticity disorder and the reorganization of neural activity could bring benefits in the clinical management of the symptom.

## LIMITATIONS OF THE STUDY

In this study, the lack of statistically significant differences in the latency and amplitude values of the cortical potentials (P1, N1, P2 and N2) and in the amplitude and duration values of the P300 between the groups can be explained by the small sample size. The reduction in the number of participants in the sample is justified by the various exclusion criteria established in this study (age, schooling, cognition, central auditory processing and hearing acuity), which could influence the LLAEP-verbal.

For future studies, we suggest measuring peripheral hearing acuity using high-frequency audiometry, since many patients with tinnitus may have alterations in these cochlear regions.

## CONCLUSION

It was possible to obtain evidence regarding the functioning of the central auditory pathway in subjects with tinnitus disorder. It was found that these subjects have a longer neural response time, which suggests disorganization of central auditory functioning. These findings reflect the possibility of the LLAEP-verbal being an additional test in the investigation of the symptom. In addition, this assessment can be a therapeutic biomarker, helping in the choice of intervention used and measurement of the interventional effects.
