# Supplementary material for: Response time, amplitude, and neural auditory maintenance in individuals with tinnitus: a comparative study
Source: Codas. 2025 Jul 28;37(4):e20240211. doi: 10.1590/2317-1782/e20240211en (PMC12315702; doi:10.1590/2317-1782/e20240211en)
Supplement: Translation

# Translation: Tempo de resposta, amplitude e manutenção auditiva neural em indivíduos com zumbido: um estudo de comparação

**DOI:** 10.1590/2317-1782/e20240211pt

**Authors:** Christine Grellmann Schumacher, Helinton Goulart Moreira, Denis Altieri de Oliveira Moraes, Larianny Rutzen Lazzari, Michele Vargas Garcia, Dayane Domeneghini Didoné

**Affiliations:** 1 Departamento de Fonoaudiologia, Centro de Ciências da Saúde, Universidade Federal de Santa Maria – UFSM - Santa Maria (RS), Brasil.

**Keywords:** Zumbido, Potenciais Evocados P300, Potenciais Evocados Auditivos, Audição, Transtornos da Audição

## Abstract

**Objetivo:**

Verificar e comparar o tempo de resposta, a amplitude e manutenção auditiva neural da via auditiva central em sujeitos com e sem transtorno do zumbido.

**Método:**

Estudo de caráter analítico, transversal e quantitativo, aprovado pelo Comitê de Ética em Pesquisa. Foram comparadas as respostas da avaliação dos Potenciais Evocados Auditivos de Longa Latência (PEALL) com estímulo verbal de 16 sujeitos com transtorno do zumbido (Grupo Estudo - GE) e 12 sujeitos sem zumbido (Grupo Controle - GC). O tempo de resposta neural foi avaliado por meio da latência dos potenciais P1, N1, P2, N2 e P300. Analisou-se também a amplitude desses potenciais. A manutenção da resposta neural foi verificada por meio dos valores de duração do componente P300. Os resultados foram comparados entre os grupos, considerando o nível de significância de 5%.

**Resultados:**

Na comparação entre os grupos dos valores de latência e amplitude dos potenciais corticais (P1,N1,P2 e N2), não foram observadas diferenças estatisticamente significantes (p>0,05). Já em relação à latência, amplitude e duração do componente P300 entre os grupos, observou-se diferença estatisticamente significante para a variável latência, sendo a mesma maior para os indivíduos com transtorno do zumbido (p<0,05).

**Conclusão:**

Indivíduos com transtorno do zumbido apresentam tempo de resposta neural maior do componente P300, o que sugere desorganização do funcionamento auditivo central.

## INTRODUÇÃO

O sistema auditivo é composto por estruturas sensoriais e conexões centrais, que tem como finalidade converter estímulos sonoros em sensações auditivas no córtex cerebral. Qualquer alteração neste sistema pode acarretar em mudanças nas diversas áreas cerebrais, o que pode ocasionar o zumbido^(1,2)^.

Atualmente, o conceito de transtorno do zumbido vai além da percepção do sintoma, mas define também, os prejuízos na qualidade de vida, devido aos impactos percebidos^(3)^. É frequente observar que, em conjunto com o zumbido, surgem queixas relacionadas à dificuldade de compreensão da fala em ambientes ruidosos e a prejuízos cognitivos, devido ao sinal neural alterado que é enviado através das conexões neurais assistemáticas que o zumbido ocasiona^(2)^. Essas manifestações podem resultar em problemas de atenção, concentração, alteração do sono, ansiedade e depressão. Este transtorno é caracterizado por uma variedade de fatores geradores e amplificadores do sistema auditivo, os quais podem ser desencadeados por alterações na organização neural da região do córtex auditivo. Essas áreas corticais são responsáveis pelo processamento auditivo, mas também estão associadas com aspectos cognitivos como memória e emoções^(4,5)^.

A partir dessa definição, Sadeghijam et al.^(2)^, descreveram a teoria do caos, resultante do funcionamento dinâmico e não linear do sistema auditivo central por conta do transtorno do zumbido. Dessa forma, ficou posto o conceito de desaferentação neural, uma alteração decorrente de qualquer diminuição de *input* auditivo ou do desequilíbrio entre excitação e inibição, que desperta um mecanismo compensatório e converte-se em uma amplificação da atividade neural espontânea e síncrona.

Para avaliar o funcionamento da via auditiva central, pode-se utilizar testes eletrofisiológicos que verificam o funcionamento e a integridade auditiva, a partir de traçados que demonstram a atividade bioelétrica das vias auditivas após estimulação acústica^(6-8)^. Dentre os testes eletrofisiológicos existentes para a avaliação do zumbido podem ser citados os Potenciais Evocados Auditivos de Longa Latência (PEALL)^(9,10)^.

Os PEALL refletem a funcionalidade do processamento auditivo central por meio da latência e amplitude dos potenciais P1, N1, P2, N2 e P300. Os componentes P1, N1 e P2 independem da resposta do sujeito e estão relacionados à integridade da via auditiva, codificação neural, percepção, detecção do estímulo e discriminação auditiva^(7)^. O N2 é considerado um componente misto, pois é desencadeado tanto por fatores endógenos quanto exógenos, contribuindo para atividades de discriminação auditiva do estímulo e refletindo fatores atencionais do indivíduo^(8)^. Já o P300 é considerado um potencial endógeno, pois depende da resposta do indivíduo e, a partir disso, fornece informação sobre a discriminação auditiva, atenção e memória recente^(7)^. Para mais, esse potencial é considerado um biomarcador para o transtorno do zumbido, já que este sintoma pode provocar mudanças no funcionamento neural auditivo e cognitivo^(9-12)^.

Alguns pesquisadores^(10)^ referem alteração nos valores de latência e amplitude dos PEALL em sujeitos com zumbido, devido ao foco de atenção fantasma que existe no sintoma, recrutando a capacidade cognitiva, como um estímulo competitivo que atrai a atenção para o sintoma^(7,9,11)^. Dessa forma, esses indivíduos podem apresentar modificação no tempo de processamento e recrutamento neural^(13)^. Ainda, pode ser observada alteração do componente P300 nesta população devido às modificações em regiões não auditivas já citadas anteriormente, as quais participam da geração desse componente^(12,14)^. Porém, pesquisadores identificaram que o zumbido crônico leve em indivíduos com audição normal não interfere na atenção auditiva dividida e na memória auditiva verbal^(11)^, ressaltando a diferenciação do conceito de zumbido e transtorno do zumbido e sua influência nos aspectos cognitivos^(3)^.

Além das medidas de latência e amplitude, outro parâmetro observável no traçado eletrofisiológico é a duração do componente P300. Hall^(15)^ traz como definição de duração a diferença em milissegundos entre a subida e a descida dessa onda, ou seja, quantos milissegundos perdura a onda P300, sendo medida desde o começo do pico até o seu final. A duração do P300 pode ser um dado importante para verificar a funcionalidade e associação de áreas auditivas e cognitivas, já que essa medida pode estar associada à quantidade de neurônios e sinapses envolvidos na geração e manutenção da atividade auditiva durante a geração desse potencial. Apesar disso, não há descrição na literatura sobre a análise e mensuração dessa variável para sujeitos com transtorno do zumbido.

Tendo como base as possibilidades de alterações auditivas centrais observadas nos componentes dos PEALL em pacientes com transtorno do zumbido e a escassez de estudos que analisem a duração do componente P300, esse estudo teve como objetivo verificar e comparar o tempo de resposta, amplitude e manutenção auditiva neural da via auditiva central em sujeitos com e sem transtorno do zumbido.

## MÉTODO

Trata-se de um estudo de caráter analítico, transversal e quantitativo, aprovado pelo Comitê de Ética em Pesquisa, sob número 64696022.1.0000.5346. A amostra foi coletada por conveniência. O contato foi realizado por meio de divulgação em redes sociais do serviço de audiologia em que a pesquisa foi realizada. Os procedimentos da pesquisa foram realizados em uma clínica escola da Universidade Federal de Santa Maria, no período de junho de 2023 a janeiro de 2024. O estudo seguiu as normas e diretrizes da Resolução 466/12 do Conselho Nacional de Saúde e todos os indivíduos que consentiram em participar da pesquisa assinaram o Termo de Consentimento Livre e Esclarecido (TCLE), em que constava a descrição dos procedimentos, riscos, benefícios e confidencialidade dos dados.

Os critérios de elegibilidade estabelecidos para os sujeitos sem e com transtorno do zumbido foram:

- idades entre 18 e 55 anos;
- ambos os sexos;
- português brasileiro como língua materna;
- preferência manual direita;
- escolarizados - mais de doze anos de escolaridade;
- limiares auditivos até 19 dBNA nas frequências de 250Hz a 8000Hz;
- curvas timpanométricas do tipo “A”, segundo Jerger et al.^(16)^;
- reflexos acústicos contralaterais presentes em níveis normais bilateralmente segundo Jerger et al.^(16)^;
  -normalidade no Instrumento de Avaliação Neuropsicológica Breve-NEUPSILIN;
- integridade no Potencial Evocado Auditivo de Tronco Encefálico (PEATE), segundo Webster (2016);
- normalidade na avaliação do Processamento Auditivo Central (PAC).

Já os critérios de exclusão, foram:

- musicistas ou expostos a prática musical;
- apresentar queixa ou comprometimento neurológico e/ou psiquiátrico diagnosticados e/ou evidentes;
- queixas de tontura ou exposição contínua ao ruído.

Para os sujeitos com transtorno do zumbido, foram acrescidos os seguintes critérios de inclusão:

- zumbido subjetivo bilateralmente, sem indícios de componente vascular (do tipo pulsátil);
- queixa de impacto na qualidade de vida;
- nota na Escala Visual Analógica (EVA) de no mínimo 4 pontos, considerando um incômodo moderado;
- tempo de percepção do sintoma maior que seis meses.

Para os sujeitos com transtorno do zumbido adotou-se os seguintes critérios de exclusão:

- realizar outra intervenção para o sintoma;
- fazer uso de medicação contínua ou tratamento farmacológico para o zumbido;

A pesquisa foi subdividida em duas etapas: no primeiro dia foram realizados os procedimentos de avaliação, sendo os procedimentos: avaliação audiológica básica, testes de processamento auditivo central e de avaliação para mensuração do zumbido (para composição inicial da casuística), totalizando uma hora e 30 minutos de duração. No segundo dia, foram realizados os procedimentos de pesquisa, sendo os PEALL (para análise dos dados), perfazendo o tempo total de uma hora e 30 minutos para a sua finalização. Estes procedimentos sempre foram realizados na mesma ordem em ambos os grupos.

Os procedimentos foram realizados para garantir a audição periférica normal, a integridade das estruturas até nível de tronco encefálico e normalidade das habilidades de processamento auditivo central, garantindo que não houvesse influência nos achados dos PEALL. Como forma de garantia da qualidade dos procedimentos realizados, ressalta-se que todos os equipamentos utilizados neste estudo estavam calibrados.

Para composição amostral, foram realizados os seguintes procedimentos: anamnese semi-estruturada, avaliação audiológica básica, inspeção visual do meato acústico externo, audiometria tonal liminar (ATL), logoaudiometria e medidas de imitância acústica, a fim de selecionar os indivíduos que atendiam aos critérios de elegibilidade atribuídos neste estudo.

No total foram atendidos 85 sujeitos, sendo 50 (58,82%) indivíduos com queixa de zumbido de ambos os sexos. Destes, seis (12%) foram excluídos por otite e/ou disfunção da tuba auditiva, 10 (20%) por doenças neurológicas e/ou psiquiátricas, seis (12%) por já estarem realizando algum tratamento para o zumbido e 12 (24%) por terem perda auditiva diagnosticada entre as frequências de 250 a 8000Hz. Desse modo, a casuística total do grupo estudo foi composta por 16 indivíduos. Já para o grupo controle, foram atendidos 35 sujeitos que não tinham queixa de zumbido. Destes, 13 (37,14%) tinham alteração de processamento auditivo central e 10 (28,57%) tinham perda auditiva, contabilizando 12 sujeitos no grupo sem transtorno do zumbido.

Os testes de PAC foram selecionados com o objetivo de contemplar a bateria mínima sugerida de acordo com as recomendações da *American Speech-Language-Hearing Association - ASHA* ^(17)^, em que um teste alterado foi considerado como Transtorno do Processamento Auditivo Central (TPAC), com base no estudo que analisou o processamento auditivo central de sujeitos com e sem zumbido^(18)^. Todos os testes foram realizados com apresentação em 40 dBNS acima da média tritonal, tendo em vista a possibilidade da utilização da mesma técnica do IPRF, uma vez que os sujeitos não apresentavam redução da acuidade periférica auditiva^(19,20)^.

Para a avaliação do PAC, os testes foram realizados em cabine acusticamente tratada, com o uso de fones supra-aurais, modelo TDH39, marca Telephonics, audiômetro de dois canais, modelo AD629B, marca Interacoustics, conectado a um *notebook* para direcionamento das avaliações. Foram realizadas as seguintes avaliações:

Teste Dicótico de Dígitos (TDD)^(20)^: Teste que avalia a habilidade de figura-fundo para sons verbais, sendo pesquisada a etapa de integração binaural. Os estímulos foram apresentados de forma binaural e foram considerados valores de normalidade iguais ou superiores a 95%^(20)^.

Teste Padrão de Frequência (TPF) Auditec^(20)^: Teste que avalia a habilidade de ordenação temporal para sons não-verbais. Os estímulos foram apresentados de forma monoaural, e utilizado o valor de referência igual ou superior a 86,6%^(20)^.

Masking Level Difference (MLD)^(20)^: Teste que analisa a habilidade auditiva de interação binaural e de atenção seletiva. Os estímulos foram apresentados de forma binaural e considerou-se como critério de normalidade o resultado de 8 dB ou mais^(20)^.

Gaps in Noise (GIN)^(21)^: Teste que avalia a habilidade de resolução temporal. Apresentou-se os estímulos de forma monoaural. Utilizou-se a faixa 1 em ambas as orelhas e adotou-se o critério de normalidade de 6ms^(21)^.

Fala no Ruído (FR)^(22)^: Teste que avalia a habilidade de fechamento auditivo para sons verbais. Os estímulos foram apresentados de modo ipsilateral com relação sinal-ruído de + 5dB. O padrão de normalidade adotado foi de 70% acertos em ambas as orelhas^(22)^.

Para garantir a preservação das habilidades neuropsicológicas, foi aplicado o NEUPSILIN^(23)^. Este protocolo, analisa oito funções cognitivas principais: orientação têmporo-espacial, atenção concentrada auditiva, percepção visual, memória, habilidades aritméticas, linguagem, praxias e funções executivas. Para o estudo, considerou-se o escore total das tarefas com os valores de normalidade sugeridos pelos autores^(23)^.

A mensuração e avaliação do transtorno do zumbido foi realizada por meio da anamnese do zumbido, com perguntas sobre o histórico de saúde geral e fatores de influência no sintoma, e por meio da Escala Visual Analógica (EVA) e do *Tinnitus Handicap Inventory* (THI).

A EVA foi impressa e numerada de 0 a 10, sendo que uma extremidade da linha significava “nenhum zumbido” e a outra “pior zumbido que se possa imaginar”. Solicitou-se que o indivíduo avaliasse o incômodo do zumbido no momento da coleta dos dados. Considerou-se incômodo moderado, nota da escala a partir de 4 pontos^(24)^.

O questionário THI foi aplicado em todos os indivíduos com transtorno do zumbido e teve como objetivo verificar a qualidade de vida e classificá-la em graus de acordo com a pontuação do teste^(25)^. O questionário possui 25 questões e cada resposta possui um valor a ser contabilizado, sendo que ao final do teste foi realizada uma somatória para verificar a pontuação e classificá-la quanto ao grau de incômodo e impacto na qualidade de vida.

Para os potenciais evocados auditivos, utilizou-se o equipamento modelo *Smart EP da marca Intelligent Hearing Systems (IHS)*, de dois canais. Inicialmente realizou-se a limpeza de pele com gel esfoliante da marca *Nuprep* e, após, os eletrodos foram fixados, sendo o eletrodo terra em Fpz; eletrodo ativo em Fz para pesquisa dos Potenciais Evocados Auditivos de Tronco Encefálico (PEATE) e em Cz (vértex craniano) para pesquisa dos PEALL. Os eletrodos de referência foram fixados nos lóbulos das orelhas direita e esquerda (A2 e A1). Utilizou-se pasta eletrolítica da marca Neurobase e os eletrodos foram fixados na pele dos indivíduos com fita micropore em pontos específicos para a realização de cada potencial. A impedância elétrica intereletrodos foi mantida igual ou menor a 3kΩ durante a realização de todas as avaliações. Para evitar maiores interferências elétricas e/ou artefatos musculares, a luz da sala foi desligada e os indivíduos foram orientados a fechar os olhos, manter-se relaxados e evitar movimentos.

O PEATE foi realizado de forma monaural, com estímulo clique de polaridade rarefeita, na intensidade de 80 dBnNA, velocidade de 27.7/s, ganho de 100K, filtro de 100-3000Hz, janela de registro de 12ms, janela do eletroencefalograma (EEG) de 31% e 2048 estímulos, sendo realizadas duas coletas para checar a reprodutibilidade das ondas. O critério para identificação da integridade da via auditiva foi a presença das ondas I, III e V e latências absolutas e intervalos interpicos dentro dos padrões de normalidade. Os valores foram analisados utilizando como referência o estudo de Webster^(26)^, levando em consideração dois desvios padrões (2 DP).

Como procedimento de pesquisa, foi realizado o Potencial Evocado Auditivo de Longa Latência com estímulo verbal (PEALL-verbal)^(27)^. Este foi realizado de forma binaural, por meio de fones de inserção na intensidade de 80 dBnNA, com 300 estímulos verbais, produzidos a partir das sílabas /ba/ e /di/, representando respectivamente, o estímulo frequente (80% das vezes) e o estímulo raro (20% das vezes) com base no paradigma oddball. No primeiro momento, realizou-se simulação do teste por meio da emissão oral da sequência /ba/ e /di/ para a compreensão dos sujeitos quanto ao funcionamento da avaliação. Em seguida, os sujeitos foram orientados a contar mentalmente o estímulo /di/. Ao final do teste o examinador perguntou quantos estímulos foram contados para comparar ao total de alvos apresentados pelo equipamento, garantindo que o indivíduo realizasse de maneira correta a atividade proposta.

A avaliação foi realizada com velocidade de 1,10/s, janela de registro de 510ms, ganho de 100K, filtro de 100-3000Hz e janela do eletroencefalograma (EEG) de 31%. Foi realizada a marcação da latência (ms) e amplitude (µs) das ondas P1, N1, P2, N2 e P300, no traçado raro^(28)^. Além disso, também foi realizada a marcação da duração da onda P300, medida em milissegundos, desde a subida até a descida do potencial (Figura 1), sendo subtraída a latência final pela inicial. Os componentes N1 e N2, foram marcados apenas quando a amplitude estava negativa.

**Figura 1:** Marcação das ondas do PEALL-verbal no traçado raro

**Fonte**: Imagem criada pelos autores, baseado na marcação dos componentes no traçado raro

Na Figura 1 apresenta-se uma imagem representativa do PEALL-verbal demonstrando a mensuração dos parâmetros analisados (latência, amplitude e duração).

Ao atender aos critérios de inclusão, os participantes da pesquisa foram distribuídos em dois grupos: Grupo Estudo (GE), composto por sujeitos com transtorno do zumbido e Grupo Controle (GC), por indivíduos sem transtorno do zumbido.

A Tabela 1 apresenta a descrição dos participantes em cada grupo quanto ao sexo e à idade. A nota da EVA média foi de 6,81 pontos (mínima= 4/ máxima= 10) no grupo com transtorno do zumbido. Ainda, o THI demonstrou pontuação média de 51 pontos (grau moderado) (mínima= 20/ máxima= 94).

**Tabela 1:** Dados descritivos da amostra

**Variável**	**GC**	**GE**	**p-valor**
n	12	16	
Sexo M/F	3 / 9	6 / 10	0,496
Idade (média)	23,83±4,69	35,75±13,48	0,004
Escolaridade	13,25±2,70	12,31±1,54	0,944

Utilizado o teste U de Mann-whitney

**Legenda:** N = tamanho da casuística; M = masculino; F = feminino; GC = grupo controle; GE = grupo estudo

Os dados foram alocados em planilha do *Excel* e as análises estatísticas foram realizadas no *software* R^(29)^ por um profissional da área. Inicialmente foi utilizado o teste Shapiro-Wilk para verificar a normalidade dos dados e, consequentemente, escolha do teste estatístico. Após, realizou-se a comparação entre orelhas em cada grupo com o teste T para amostras pareadas. A análise entre os grupos foi realizada por meio do teste T para amostras independentes. Considerou-se o nível de significância de 5% para todas as análises realizadas.

## RESULTADOS

Inicialmente os resultados foram analisados entre o lado direito e esquerdo intra-grupos. Não foi observada diferença estatisticamente significante (p>0,05) entre os lados para todas as variáveis analisadas. Dessa forma, considerou-se a média dos valores para comparação entre os grupos.

Não foi observada diferença estatisticamente significante na comparação dos valores de latência e amplitude dos potenciais P1, N1, P2 e N2 entre os grupos (Tabela 2).

**Tabela 2:** Comparação dos valores de latência e amplitude dos potenciais P1, N1, P2 e N2 entre os grupos

**Variável**	**Grupo**	**n**	**Média ± DP**	**Mín - Máx**	**Diferença**	**P-valor**
Latência P1	GC	12	69,5 ± 12,21	54 - 95	4,59	0,362
	GE	16	64,91 ± 13,51	45 - 85		
Amplitude P1	GC	12	4,3 ± 1,59	1,0 - 6,9	0,07	0,938
	GE	16	4,23 ± 2,67	0,3 - 10,9		
Latência N1	GC	12	114,21 ± 10,49	94,0 - 148,0	0,43	0,929
	GE	16	113,78 ± 13,68	89,0 - 145,0		
Amplitude N1	GC	12	-5,02 ± 1,47	1,9 - 8,5	0,5	0,686
	GE	16	-5,52 ± 4,58	4,9 - 13,4		
Latência P2	GC	12	179,88 ± 14,61	156,0 - 205,0	11,34	0,145
	GE	16	191,22 ± 22,83	141,0 - 239,0		
Amplitude P2	GC	12	4,4 ± 3,13	0,9 - 11,9	0,15	0,893
	GE	16	4,24 ± 2,85	0,4 - 10,9		
Latência N2	GC	12	249,82 ± 33,72	205,0 - 304,0	14,15	0,318
	GE	16	263,97 ± 36,56	145,0 - 322,0		
Amplitude N2	GC	12	-2,98 ± 2,14	0,4 - 7,7	1,1	0,257
	GE	16	-1,87 ± 2,61	3,3 - 7,5		

Utilizado Teste T para amostras independente

**Legenda:** n = tamanho da casuística; L = latência; A = amplitude; GC = grupo controle; GE = grupo estudo

Na comparação dos valores de latência, amplitude e duração do componente P300 entre os grupos (Tabela 3), observou-se diferença estatisticamente significante para a variável latência, sendo a mesma maior para os indivíduos com transtorno do zumbido. As variáveis amplitude e duração apresentaram um valor de significância abaixo de 10%, indicando uma possível tendência à diferença entre os grupos.

**Tabela 3:** Comparação dos valores de latência, amplitude e duração do potencial P300 entre os grupos

**Variável**	**Grupo**	**n**	**Média ± DP**	**Mín - Máx**	**Diferença**	**P-valor**
**Latência P3**	GC	12	306,91 ± 40,99	226,0 - 373,0	34,53	**0,021**
	GE	16	341,44 ± 31,91	256,0 - 418,0		
Amplitude P3	GC	12	5,17 ± 3,53	1,3 - 9,6	1,93	0,085
	GE	16	3,24 ± 2,07	0,7 - 9,1		
Duração P3	GC	12	132,17 ± 42,09	63,0 - 215,0	29,07	0,076
	GE	16	103,09 ± 40,63	21,0 - 186,0		

Utilizado Teste T para amostras independente

**Legenda:** n = tamanho da casuística; L = latência; A = amplitude; GC = grupo controle; GE = grupo estudo

Na Figura 2 encontra-se a representação gráfica dos PEALL-verbal para os grupos controle e zumbido, com diferença estatisticamente significante apenas para latência de P300.

**Figura 2:** Representação gráfica do PEALL-verbal entre os sujeitos com e sem transtorno do zumbido

**Fonte:** Criada pelos pesquisadores

**Legenda:** GC = grupo controle; GE = grupo estudo; ** Diferença estatisticamente significante para a latência do P300

## DISCUSSÃO

Os potenciais evocados auditivos são utilizados para detectar a atividade neuronal e a ativação das fibras auditivas, sendo de suma importância a compreensão da neurofisiologia do zumbido para o processo terapêutico. A realização do PEALL-verbal como biomarcador em pacientes com zumbido traz grandes achados quanto à influência do sintoma em aspectos auditivos centrais^(10)^. Este potencial tem demonstrado cada vez mais sua influência clínica, revelando as capacidades cognitivas e de habilidades auditivas por meio dos componentes avaliados e seus sítios geradores. Sendo assim, se torna um aliado na avaliação clínica, para mensuração dos impactos do zumbido, levando em conta a manifestação do sintoma e mudanças no funcionamento auditivo-cognitivo-emocional-executivo, das múltiplas áreas cerebrais.

Este estudo vai ao encontro de pesquisas internacionais recentes, como a de Cardon et al.^(30)^, que objetivaram desenvolver um modelo para detecção de casos de zumbido baseado nos potenciais evocados auditivos como biomarcadores no diagnóstico e monitoramento. Do mesmo modo que Morse e Vander Werff^(31)^, a presente pesquisa buscou-se observar as respostas dos Potenciais Evocados Auditivos Corticais (PEAC), avaliando o início auditivo cortical *versus* respostas de deslocamento. As citações demonstram a inquestionável relevância do tema, expondo os achados dos PEALL em sujeitos com transtorno do zumbido.

Um grande diferencial deste estudo centra-se na mensuração para além da latência, objetivando demonstrar a responsividade neural (amplitude de resposta) dos componentes e a duração do componente P300. Estudos demonstram a importância da análise da duração em potenciais evocados auditivos. Um estudo pioneiro realizado em idosos demonstrou a importância dessa medida, no qual realizou a análise para o potencial P300 e para o *Mismatch Negativity* ^(32)^. Tal análise foi empregada com o objetivo de mensurar o tempo para a discriminação auditiva, já que a duração envolve a latência inicial e final do P300. Logo, levando em consideração os impactos advindos da percepção do zumbido, observar a sua manutenção neural para contagem - memória- do estímulo é de suma relevância.

Cabe destacar que os estudos recentes que objetivaram mensurar as mudanças neuroplásticas da via auditiva central, principalmente em regiões corticais auditivas, normalmente apresentavam populações não homogêneas ou com outras variáveis associadas, sejam elas relacionadas ao envelhecimento, patologias ou transtornos da percepção dos sons^(33,34)^.

Dessa forma, acredita-se que, para a presente pesquisa, a diferença da latência do P300 entre os grupos ocorreu devido à provável desorganização neural dos pacientes com transtorno do zumbido, já que as variáveis escolaridade e sexo não apresentaram diferenças estatisticamente significantes na comparação entre os grupos, ou seja, supõe-se semelhança no nível cognitivo dos pacientes. Acredita-se que, apesar da diferença da faixa etária entre os grupos, essa variável não influenciou nos resultados da latência do P300. Uma pesquisa destaca mudanças significativas nos valores de latência a partir dos 60 anos de idade^(35)^, faixa etária não incluída no presente estudo. Ainda, pesquisadores^(36)^, objetivando caracterizar a via auditiva dos idosos, evidenciou que o P300 é menos sensível para alterações da idade, mas sim por características específicas^(37)^. Deste modo, pode-se sugerir que o potencial tenha influência da percepção do sintoma e do desajuste neural ocasionado pelo zumbido, e não necessariamente da idade, tendo em vista que foram incluídos apenas adultos de alta escolaridade.

Em relação aos potenciais auditivos corticais, os dados da presente pesquisa não apontaram diferenças entre os grupos para os potenciais P1, N1, P2 e N2, como já observado em outros estudos^(38)^. Alguns pesquisadores observaram diferenças apenas para a amplitude do componente P2, fato que pode estar relacionado com a influência do zumbido na discriminação dos estímulos acústicos apresentados.

A partir da análise dos resultados dos potenciais P1, N1, P2 e N2, descritas na Tabela 1, foi observado que os indivíduos sem e com zumbido possuem funcionalidade semelhante das regiões auditivas corticais. Uma revisão sistemática publicada recentemente^(9)^, destacou que os pacientes com zumbido apresentaram alterações do funcionamento das vias auditivas centrais, sendo constatado alterações nos valores de latência e/ou amplitude dos potenciais de longa latência relacionados a eventos. Contudo, os autores destacam que essas alterações estão comumente associadas à gravidade do zumbido, local da lesão e a capacidade de alterações após as intervenções. Tal afirmação sobre estas mudanças ainda são incongruentes na literatura especializada, já que estudos demonstram mudanças relevantes e outras semelhanças no funcionamento auditivo cortical de pacientes sem e com zumbido^(9,10,31)^.

Indivíduos com zumbido comumente apresentam alterações nas habilidades do PAC. No entanto, na presente pesquisa, os participantes com a percepção do sintoma não possuíam queixas e as habilidades mantiveram-se dentro dos padrões de referência. Dessa forma, esse pode ser outro aspecto que justifica os achados semelhantes dos componentes P1, N1, P2 e N2 entre o grupo controle e grupo estudo, pois estes potenciais representam a funcionalidade central de diversas habilidades auditivas, que quando alteradas podem influenciar nas variáveis latência e amplitude desses componentes^(10,18)^.

Os achados da presente pesquisa evidenciaram alteração na latência do potencial P300 quando comparados sujeitos sem e com transtorno do zumbido. Os sujeitos com transtorno do zumbido apresentam uma desaferentação de estímulos na região talâmica, com hiperativação em região parietal e giro temporal, devido ao mecanismo fisiopatológico^(39,40)^. Assim, pode-se esperar alterações nas regiões centrais, as quais são sítios geradores do componente P300. Esse achado também foi observado em outras pesquisas, no qual observou-se alteração nos valores de latência do P300^(14)^. Uma recente revisão sistemática com meta-análise^(30)^ destacou que o P300 é o principal biomarcador para sujeitos com zumbido dentre os PEALL, evidenciando a importância do achado em relação à latência do P300 no presente estudo.

Cabe destacar que pesquisas^(41)^ com sujeitos com zumbido há menos de 10 meses, não evidenciam diferenças significativas para as características do P300, ressaltando a relação da diferenciação de zumbido e transtorno do zumbido, proposta por De Ridder^(3)^, ou seja, o tempo da percepção do sintoma está relacionado com maior desorganização neural e outros efeitos cognitivos e psicológicos. Na presente pesquisa, foram incluídos apenas sujeitos com transtorno do zumbido, caracterizado por um incômodo moderado, sugerindo que há uma modificação da via auditiva central em sujeitos com esta característica.

De modo geral, o transtorno do zumbido interfere principalmente nos aspectos relacionados à atenção, memória, percepção de fala e diretamente na qualidade de vida, ou seja, no comportamento do indivíduo. Os potenciais P1, N1, P2 são corticais, endógenos e “automáticos”. O P2 é considerado importante para observar a disritmia talâmica ou inibição central^(33)^. No entanto,a sensibilidade desse componente para sujeitos com zumbido é controversa na literatura. Acredita-se, desse modo, que os componentes N2 e P300 possam refletir com maior qualidade o comportamento do indivíduo relacionado ao sintoma^(38)^. Logo, as diferenças nestes potenciais em sujeitos com zumbido são mais comuns, conforme foi observado na latência do P300 no presente estudo (Figura 2).

A amplitude do P300 está intrinsecamente relacionada à ativação neuronal para responder a atividade proposta para gerar este componente. Sujeitos com zumbido, devido a arritmia talâmica, podem apresentar uma inibição na atenção do estímulo externo, gerando uma amplitude menor neste componente^(9)^. Apesar de no presente estudo não ter sido evidenciada diferença estatisticamente significante, observou-se possível tendência à diferenças entre os grupos (p-valor abaixo de 10%), sendo os valores reduzidos nos pacientes com transtorno do zumbido. A grande variabilidade nos valores de amplitude e o reduzido tamanho amostral podem ter contribuído para esses resultados não terem sido estatisticamente significantes.

A duração do componente P300 está associada à manutenção da atividade neuronal durante a tarefa auditiva^(9,37)^. Neste estudo, da mesma forma que a amplitude, foi evidenciada uma possível tendência estatisticamente significante entre os grupos, sendo que os resultados foram menores no grupo de sujeitos com zumbido, mas não apresentaram significância. Esses dados chamam atenção, pois notou-se duração reduzida em sujeitos com transtorno do zumbido e processamento auditivo normal, sugerindo que a percepção deste sintoma pode influenciar diretamente no funcionamento neuronal destes sujeitos. Acredita-se que um número amostral maior poderia evidenciar diferenças significativas entre os indivíduos avaliados. Não foram evidenciados estudos que descrevessem sobre a duração do potencial P300 em sujeitos sem e com transtorno do zumbido, o que traz ineditismo à presente pesquisa.

A literatura sugere a utilização de diferentes tipos de estímulos para avaliação dos PEALL em indivíduos com queixa de zumbido, incluindo os estímulos de fala utilizados nesta pesquisa. Esses estímulos são ideais para verificar a funcionalidade das bases neurais da detecção e discriminação da fala, contribuindo para informações adicionais do processamento de sinais complexos^(10,27)^. É importante ressaltar que nesse tipo de avaliação, o zumbido pode se tornar um terceiro estimulo auditivo^(11,42)^, influenciando nos componentes dos PEALL, principalmente no P300, o qual esta associado a aspectos de discriminação auditiva, atenção e memória. Dessa forma, o aumento nos valores de latência e redução dos valores de amplitude e duração do P300 dos sujeitos da presente pesquisa podem ser justificados à esse possível desvio atencional, ou seja, o recrutamento e manutenção neural para o estímulo raro da avaliação dos PEALL tornou-se mais difícil devido à presença do zumbido, impactando diretamente na velocidade de processamento neural.

O fato deste estudo evidenciar alteração no tempo de resposta auditiva e sugerir alterações no recrutamento e manutenção neural evidenciados por meio da latência, amplitude e duração do componente P300 traz um novo olhar para a utilização clínica do PEALL em sujeitos com transtorno do zumbido, destacando a importância das análises citadas para verificar a manutenção da atividade neuronal durante o processo auditivo e cognitivo na avaliação eletrofisiológica.

Portanto, pode ser útil aplicar esse conhecimento em terapias de zumbido baseadas em *neurofeedback*, visando maximizar a capacidade de desviar a atenção do zumbido, assim como o uso das terapias cognitivo-comportamentais para desvio do foco de atenção ao sintoma. Outro aspecto relevante está relacionado à análise do potencial P300 como um possível biomarcador no processo de reabilitação do sintoma, avaliando os aspectos antes e depois da intervenção. Considerando as áreas corticais e cognitivas representadas pelo PEALL, a estimulação da via auditiva-cognitiva com o objetivo de promover a reorganização central pode ser uma estratégia terapêutica eficaz, uma vez que se trata de um distúrbio de neuroplasticidade, e a reorganização da atividade neural pode trazer benefícios no manejo clínico do sintoma.

## LIMITAÇÕES DO ESTUDO

No presente estudo, a não observação de diferenças estatisticamente significantes nos valores de latência e amplitude dos potenciais corticais (P1, N1, P2 e N2) e nos valores de amplitude e duração do P300 entre os grupos pode ser justificada pelo reduzido tamanho amostral. A redução do número de participantes da amostra estudada justifica-se devido aos diversos critérios de exclusão estabelecidos neste estudo (idade, escolaridade, cognição, processamento auditivo central e acuidade auditiva), os quais poderiam influenciar no PEALL-verbal.

Para os estudos futuros sugere-se a realização da mensuração da acuidade auditiva periférica utilizando também a audiometria de altas frequências, já que muitos pacientes com transtorno do zumbido podem apresentar alterações nessas regiões cocleares.

## CONCLUSÃO

Foi possível obter evidências quanto ao funcionamento da via auditiva central em sujeitos com transtorno do zumbido. Constatou-se que esses sujeitos apresentam tempo de resposta neural maior, o que sugere desorganização do funcionamento auditivo central. Tais achados refletem a possibilidade do PEALL-verbal ser um exame adicional na investigação do sintoma. Além disso, esta avaliação pode ser um biomarcador terapêutico, auxiliando na escolha da intervenção utilizada e mensuração dos efeitos interventivos.
